# External features enriched model for biomedical question answering

**DOI:** 10.1186/s12859-021-04176-7

**Published:** 2021-05-26

**Authors:** Gezheng Xu, Wenge Rong, Yanmeng Wang, Yuanxin Ouyang, Zhang Xiong

**Affiliations:** 1 State Key Laboratory of Software Development Environment, Beihang University, No.37 Xueyuan Road, Beijing, 100191 China; 2 Sino-French Engineer School, Beihang University, No.37 Xueyuan Road, Beijing, 100191 China; 3 School of Computer Science and Engineering, Beihang University, No.37 Xueyuan Road, Beijing, 100191 China; 4 Ping An Technology, Xinyuannanlu No.3, Beijing, 100027 China

**Keywords:** Biomedical question answering, Feature fusion, Pre-trained language model, POS, NER

## Abstract

**Background:**

Biomedical question answering (QA) is a sub-task of natural language processing in a specific domain, which aims to answer a question in the biomedical field based on one or more related passages and can provide people with accurate healthcare-related information. Recently, a lot of approaches based on the neural network and large scale pre-trained language model have largely improved its performance. However, considering the lexical characteristics of biomedical corpus and its small scale dataset, there is still much improvement room for biomedical QA tasks.

**Results:**

Inspired by the importance of syntactic and lexical features in the biomedical corpus, we proposed a new framework to extract external features, such as part-of-speech and named-entity recognition, and fused them with the original text representation encoded by pre-trained language model, to enhance the biomedical question answering performance. Our model achieves an overall improvement of all three metrics on BioASQ 6b, 7b, and 8b factoid question answering tasks.

**Conclusions:**

The experiments on BioASQ question answering dataset demonstrated the effectiveness of our external feature-enriched framework. It is proven by the experiments conducted that external lexical and syntactic features can improve Pre-trained Language Model’s performance in biomedical domain question answering task.

## Background

Recently with the development of network technology and the accumulation of big data, more and more healthcare services have appeared, including online medical information retrieval and biomedical question answering applications, which can help people seek health information and biomedical knowledge quickly and economically [1]. Among these healthcare application scenarios, biomedical question answering technology, a sub-task of natural language processing in the biomedical domain which could locate and extract required biomedical text spans, is a basic and useful method for knowledge retrieval and representation.

Question answering (QA) task is an essential part of neural language processing (NLP), of which biomedical question answering is always an important and challenging branch. Nowadays, with the emergence of large-scale labelled question answering datasets and the development of neural network models, machine reading comprehension (MRC) based QA tasks have been widely studied. In general, the goal of MRC based QA task is to answer a specific question given one or more related passages. It could still be divided into two main sub-classes according to different methods of obtaining answers: extractive and generative. For generative QA task, the expected answer is usually not present in the given context and needs to be inferred and generated [2]; whereas for the extractive task, the answer span could be extracted from one or more precise places in the given passages [3], which is more reasonable and expected for biomedical question answering researches. It is useful and highly applicable since it could provide a reliable answer to the users among many related biomedical passages and act as the last step in the automatic biomedical QA system and some healthcare services.

Traditionally, a pipeline of question answering consists of three main steps: feature engineering, question classification and answer processing [4], where the first step is about text feature construction such as named-entity recognition (NER) and part-of-speech (POS). Since the emergence of the language model and deep neural network model, people started to leverage continuous text representation to complete QA task, in which feature engineering is still an important part. They usually keep question-answer word matching and other syntactic and lexical information as additional feature embeddings aligned with word embeddings to enhance task performance [3]. Recently, large-scale pre-trained language models (PLM) have achieved excellent performance in various NLP tasks [5, 6] including question answering.

As for biomedical question answering, considering the specific characteristics of biomedical corpora, feature engineering plays a more important role in the question-answer matching process and affects the final task performance. Researchers have made many attempts in this regard. On the one hand, they employed the unlabeled biomedical text to train a domain language model, in order to obtain a more adaptive biomedical text representation [4, 7]; on the other hand, they tried to introduce some domain features like biomedical NER to enrich the original QA text [8, 9]. However, there are still a lot of problems to be solved in BioQA task. For example, compared with general corpora, biomedical text usually contains a large number of abbreviations, domain proper nouns, and non-alphanumeric characters [10], which can hardly be all covered by the biomedical NER that merely focuses on a specific biomedical category named entity’s recognition such as disease entity or gene entity; besides, for a biomedical question like “What is the genetic basis of Ohdo syndrome?”, the start-of-the-art model’s answer is “Lujan syndrome” [11], which is far from the golden answer “mutations in MED12” as it pays too much attention to biomedical concept “syndrome” but ignores syntactic features and confuses expected answer type of question. Moreover, considering the small scale of biomedical QA dataset, inappropriate ways of adding biomedical information such as latent answer type (LAT) in the domain task fine-tuning process can sometimes affect the robustness of the original model and even result in some negative effects [12].

In this research, we focused on extractive question answering task in biomedical domain and proposed a framework to extract external syntactic and lexical features, such as POS and general NER, and to fuse these auxiliary features into the sentence representation encoded by pre-trained language model in order to enrich the model with more syntactic information, emphasize the lexical representation of biomedical text, enhance the matching degree between question and passages and bridge the representation gap between general and domain corpus without disturbing the PLM performance. We have demonstrated our idea in BioASQ 6b, 7b, 8b tasks and achieved a promising performance.

### Related work

**Biomedical Question Answering Models**

The biomedical QA task has attracted many NLP researchers’ attention in recent years due to its wide range of applications and unique domain textual characteristics. Lots of approaches and models have been proposed in the community. For example, Wiese et al. [4] proposed an RNN-based QA model, leveraging biomedical Word2Vec embeddings to realize the domain transfer learning. Nowadays, with the emergence of pre-trained language model including ELMo [6], BERT [5], XLNet [13], researchers usually use PLM structure as embedding and encoding modules, then add several concise downstream task layers to transfer the pre-trained language model to complete a specific task, such as question answering and text classification. In the biomedical domain, Lee et al. presented BioBERT [7], a large scale pre-trained language model based on BERT and trained on several biomedical corpora, including 200k PubMed abstracts, 270k PMC full texts, and a combination of these two, which leads to an obvious performance augmentation in many biomedical NLP tasks. Based on biomedical pre-trained language model, Jeong et al. [14] recently proposed to make use of transfer learning to enhance domain QA’s performance.

**External Features in general NLP Tasks**

Featuring engineering always plays an important role in machine learning. With the development of neural network framework in recent years, many efforts have been devoted to capturing external textual features and merging them into deep learning models to enhance their performance in different NLP related tasks. There are various manifestations of external features. For example, based on RNN framework, Chen et al. [3] proposed an open-domain QA model DrQA, which used lexical and semantic features like POS, NER, and question-context matching information as a part of input; Qu et al. [15] proposed a history answer embedding as the external characteristics to the original BERT embedding in the conversational question answering task. Besides, to obtain a better textual representation under PLM framework, Levine et al. [16] took advantage of lexical-semantic level information extracted by WordNet in the BERT pre-training phase; Wang et al [17] incorporated word and sentence structural features into pre-training process to enhance language understanding.

On the other hand, how to introduce these external features without influencing the robustness and performance of the original neural network model has also been widely studied. For instance, Chen et al. [3] simply aligned POS and NER features with input text as additional labels in DrQA. Wu et al. [18] emphasized the entity place information by adding ‘$’ symbol in the raw input sentence for the entity relation classification task using BERT pre-trained language model. Qu et al. [15] directly added the additional embedding information on the original BERT embeddings for the conversation QA task.

**External Features in Biomedical Question Answering Task**

Considering the domain characteristics of biomedical texts, scientific researchers have already noticed the importance of external features. For example, in RNN-based models, lexical and syntactical features play an important role in the QA task. Wiese et al. [4] added bio-entity tag embeddings as external features in extractive biomedical QA task. Besides, under the bidirectional attention flow network structure, Oita et al. [19] attempted a post-processing module to match the candidate answers with biomedical NER features in the answer selection process, which indeed improved the model’s performance but was still inferior to pre-trained language model. Lamurias et al. [8] enriched the question and answer texts using MER [9], a biomedical named entity recognition tool, for ranking and selecting type QA task. However, their attention is mainly on biomedical features and domain terminology. Nowadays, under the framework of pre-training language models, the training process involves both biomedical corpus and general texts, but not enough attention has been paid to general external features. How to select and extract meaningful external features from both of these two different domains at the same time has not been widely concerned. Besides, considering the small-scale dataset of Biomedical QA task, the choice of the added external features and the feature fusion method should be paid special attention; otherwise, it may cause negative effects. For example, Telukuntla et al. [12] introduced latent answer type (LAT) features in the biomedical QA task by adding special marks to the original question and passages text, which has realized the type distinction goal but caused a slight decline in the overall performance of the model.

Unlike the aforementioned methods which did not attach special attention to general features or which did not elaborate their usage of these features clearly, this article focuses on the general lexical and syntactical features such as POS and NER. Furthermore, based on the pre-trained language model, a new feature fusion framework is proposed to explore a reasonable method of how to use these external features, aiming to improve the performance of question answering tasks in the biomedical field.

## Methods

**Fig. 1:**
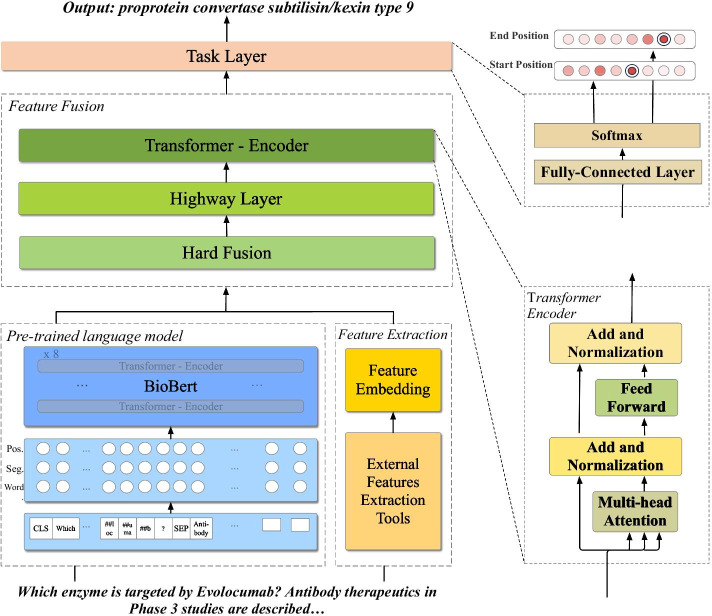
Model overall architecture. External features enriched model specific network architecture based on BERT pre-trained language model

In this section, we will explain our feature-enriched framework for biomedical question answering task. The overall architecture is shown as Fig. 1. Firstly, we will give the problem definition and the model overview. Secondly, we will introduce the usage of pre-trained language model and external feature extraction methods. Afterwards, we will present the feature fusion module and the answer selection process.

### Problem definition and model overview

For an extractive question answering task, given a context passage $P=\{x_1, \ldots , x_m\}$ and a question $Q=\{q_1, \ldots ,q_n\}$, supposing there exists one and only one answer span $A=\{x_i\}^{l_b}_{l_a}$ consisting of continuous tokens in the context passage, where $x_i$ represents context token, $q_i$ represents question token, *m* is the context passage length and *n* is the question length. Besides, $l_a$ and $l_b$ represent respectively the start and end position of answer span in context passage. The goal is to locate the answer boundary $l_a$ and $l_b$.

In this framework, we will firstly use pre-trained language model BioBERT to encode our input sentences *P* and *Q* into a sequence of continuous representation. Since BioBERT leverages the same vocabulary list and WordPiece tokenization method as BERT, although the biomedical vocabulary is quiet different from general vocabulary, the domain words will be separated into small pieces and it can largely avoid the out-of-vocabulary (OOV) phenomena. Therefore, the final input of BioBERT model is $\{[CLS],Q\prime ,[SEP],P\prime \}$ where $P\prime$ and $Q\prime$ are sub-tokenized word pieces sequence of original *P* and *Q*, and [*CSL*], [*SEP*] are BERT special marks used for separating the sentence pairs and for some classification tasks. We note output of BioBERT pre-trained language module as $H_{b}$:

1 $$\begin{aligned} H_{b}=\{{[CLS]},t_1,\ldots ,t_{n\prime },{[SEP]},t_{n\prime + 2},\ldots ,t_{m\prime }\} \end{aligned}$$

Simultaneously, the input sentences will be mapped to a sequence of tokens embedded in a continuous space by the external feature extraction module, noted as $H_{f}$, with the same form of $H_{b}$.

Afterwards, we will use a feature fusion module to merge these two sentences representations and finally send them to the specific task layer, and predict the answer span start and end position $l_a$ and $l_b$.

### External feature extraction

External features can provide various and rich information for unstructured texts. As it is better to fine-tune our PLM on both general QA dataset (SQuAD [20]), and biomedical QA dataset (BioASQ dataset) [21] for a better QA performance, we introduced POS and NER these two general textual features which can bridge the gap between the general corpus and the biomedical contexts while providing needed syntactic and lexical features.

In order to introduce and merge these semantic and lexical features in QA task, we utilized some off-the-shelf tools, including NLTK and spaCy. spaCy is a library for advanced Natural Language Processing in Python and Cython, it provides fast and convenient APIs for tasks such as tagging, parsing and named entity recognition, based on very latest research. As for natural language toolkit (NLTK) [22], it is a platform built for NLP learning, providing a lot of annotated corpora and a suite of text processing libraries for various NLP tasks.

As shown in Fig. 1, we utilized these two tools to tag the POS and NER features of input tokens and align them with BERT WordPiece tokenization result. Then, we used an embedding layer, which is a linear transformation, to map these two one-hot features, $F_{POS}$ and $F_{NER}$, to a continuous feature space, respectively. Afterwards, we concatenated these two feature embeddings as the feature extraction module output:

2 $$\begin{aligned} E_{POS}= & \, M_{POS}F_{POS}, E_{NER} = M_{POS}F_{POS} \end{aligned}$$

3 $$\begin{aligned} H_f= & \, [E_{POS}; E_{NER}] \end{aligned}$$

where $M_{POS}\in {\mathbb {R}}^{1\times m_{POS}}$ and $M_{NER}\in {\mathbb {R}}^{1\times m_{NER}}$ are trainable weights, $m_{POS}$ and $m_{NER}$ are hyperparameters, and “;” represents concatenation in the last dimension.

### Feature fusion

In this post-BERT block, we proposed a feature fusion structure to merge together extracted external features and BioBERT output, aiming to bring in the external textual information without disturbing original BERT’s stability, and then utilized a simple task layers to predict the answer span boundary. Feature fusion consists of three sub-layers, firstly, we used a hard fusion layer, extending the feature embeddings to the same dimension as BioBERT output, adding them together roughly and activating by a sigmoid function:

4 $$\begin{aligned} H_{hard} = \sigma (H_b+M_fH_f ) \end{aligned}$$

Next, we used a highway network [23] to further fuse these representations. For the feedforward non-linear transformation layer $F(\cdot )$, we utilized *tanh* as activation function; as for the transform gate $T(\cdot )$, we used sigmoid function to activate:

5 $$\begin{aligned} &H_{Highway} \\&\quad =F(H_{hard})\odot T(H_{hard}) + H_{hard}\odot (1-T(H_{hard})) \end{aligned}$$

where “$\odot$” represents the element-wise multiplication. Afterwards we leveraged a transformer-encoder [5, 24] to catch the inter-dependency between the feature-enriched tokens, as shown in Fig. 1:

6 $$\begin{aligned} H_{encoded} = {TEncoder}(H_{Highway}) \end{aligned}$$

where $H_{encoded}$ is the final output of feature fusion module.

### Answer span prediction

To complete the answer boundary prediction task, we added a simple fully-connected layer at the end and utilized softmax activation function to simulate the start and end token position’s distribution:

7 $$\begin{aligned} P_{s}^{i}= & \, \frac{exp(W_t \cdot H_{encoded}^{i})}{\sum _{k=0}^{m\prime }exp(W_t \cdot H_{encoded}^{k})} \end{aligned}$$

8 $$\begin{aligned} P_{e}^{j}= & \, \frac{exp(W_t \cdot H_{encoded}^{j})}{\sum _{k=0}^{m\prime }exp(W_t \cdot H_{encoded}^{k})} \end{aligned}$$

where $P_{s}^{i}$ represents the possibility that the *i*-th token is the answer start position, and the $m\prime$ is the total length of input sequence.

We defined average log-likelihood of start and end position as our training objective:

9 $$\begin{aligned} {\mathcal {L}}=-\frac{1}{2N}\sum _{n=1}^{N} logP_{s}^{y_{s}^{n}}+logP_{e}^{y_{e}^{n}} \end{aligned}$$

where N is the batch size and $y_{s}^{n}$, $y_{e}^{n}$ represent respectively golden answer’s start and end token index for *n*-th example.

**Table 1:** Dataset overall information

Dataset name	Raw training set	Post-processed training set	Testing set
SQuAD1.0	107,785	107,785	–
BIOASQ 6b	619	4772	161
BIOASQ 7b	779	5537	162
BIOASQ 8b	941	10147	151

## Results

### Datasets

The main data of the experiment comes from BioASQ challenge, an annual competition on large-scale biomedical semantic indexing and question answering (QA) organized since 2013 [25]. BioASQ comprises two main tasks, task A is about the annotation of new biomedical documents from PubMed, a free search engine for life science and biomedical references, with MESH headings; task B consists of several biomedical semantic QA tasks, including information retrieval, multi-type (yes/no, factoid and list) question answering, and summarization tasks. In this research, we focused mainly on factoid question answering of task B, which is the most similar branch with reading comprehension based QA task. Therefore, We employed the factoid QA datasets of 2018 (6b), 2019 (7b) and 2020 (8b) challenges to verify our model. To enhance the reliability of the comparison experiments and verify the effectiveness of our model, we directly used the pre-processed 6b, 7b^1^ and 8b^2^ training data provided by DMIS-Lab.

Besides, since the emergence of pre-trained language models, the performance of question answering tasks has been remarkably improved on a lot of large-scale general QA datasets, including SQuAD1.0 [20], a widely used general reading comprehension dataset containing more than 100k question-answer pairs posed by crowd workers on a set of Wikipedia articles^3^. However, limited by the size of training data, the performance of domain QA tasks still has room for improvement. It is verified by Gururangan et al. [26] that fine-tuning a PLM firstly on a general task-oriented dataset can help to improve model’s performance of this task on a domain dataset as well, and this methodology is widely used in biomedical natural language processing tasks. Therefore, in our experiments, we utilized SQuAD1.0 [20] to firstly fine-tune our model and to promise the performance. Table 1 shows some basic statistical information about the general and biomedical datasets mentioned above, from which we could notice the huge distance between biomedical QA datasets and general QA dataset in number.

### Configuration and training details

In the experiment, we mainly utilized BioBERT parameters to initialize our network and fine-tuned the model sequentially in SQuAD and biomedical training set. As we mentioned in “Methods” section, we employed two off-the-shelf tools, NLTK and spaCy, to extract part-of-speech (POS) and named-entity recognition (NER) features from unstructured question and passages. We have kept all of the 36 part-of-speech tags and chosen 12 commonest named-entity tags that appeared in the biomedical text, including *PERSON, NORP, ORG, DATE, TIME, PERCENT, GPE, PRODUCT, QUANTITY, ORDINAL and CARDINAL*. Regarding padding tokens and BERT marks as two independent classes, we set 38 and 14 as hyper-parameters for embedding dimensions in the feature extraction module. The parameters of BioBERT pre-trained language model, feature embedding module, feature fusion module, and task layers are all trainable. To avoid the contingency of the experimental results and to verify the robustness of the model, we chose different seeds (12345, 24, 488) randomly to repeat the experiments, and the average results are shown in Table 4. Other than that, the other tables’ results (in Tables 2, 3 and 5) are the optimal experimental results among these three seeds.

Besides, to further compare and verify the effectiveness of the proposed model, we also conducted two contrast tests using BERN biomedical named entity extractor [27] and SciBERT pre-trained language model [28] respectively. BERN APIs could extract seven different categories of biomedical named-entity from a free passage, including gene, disease, drug, specie, mutation, miRNA and pathway. SciBERT is a BERT based PLM trained on scientific texts containing biomedical corpus.

All experiments are compiled and tested on a Linux server (CPU: Intel(R) Xeon(R) CPU E5-2678 V3 @ 2.50GHz; GPU: NVIDIA GeForce RTX 2080Ti). We trained our model with a relatively small batch size of 8.

**Table 2:** Comparison of best experimental results on BioASQ 6b, 7b and 8b

Model	6b Factoid QA			7b Factoid QA			8b Factoid QA		
	SAcc	LAcc	MRR	SAcc	LAcc	MRR	SAcc	LAcc	MRR
AUTH [29]	0.2015	0.4020	0.2713	0.2363	0.3710	0.2898	0.1642	0.2853	0.2105
ZhuLab-Fudan [30]	0.2387	0.3314	0.2762	0.2765	0.3922	0.3252	0.3509	0.5141	0.4115
Google [31]	–	–	–	0.4201	0.5822	0.4798	–	–	–
BioBERT [11]	0.4286	0.5714	0.4841	0.4367	0.6274	0.5115	–	–	–
UNCC [12]	–	–	–	0.3554	0.4922	0.4063	–	–	–
Umass [32]	–	–	–	–	–	–	0.3133	0.4798	0.3780
KU-DMIS-2020 [14]	0.4141	0.6134	0.4805	**0**.**4510**	0.6245	0.5163	0.3819	0.5719	0.4593
Our Model	**0**.**4517**	**0**.**6294**	**0**.**5197**	0.4444	**0**.**6419**	**0**.**5165**	**0**.**3937**	**0**.**6098**	**0**.**4688**

Bold values represent the highest results

**Table 3:** Ablation experiment results of our model

Model	6b Factoid QA			7b Factoid QA			8b Factoid QA		
	Sacc	Lacc	MRR	Sacc	Lacc	MRR	Sacc	Lacc	MRR
Baseline (BioBERT)	0.3973	0.6217	0.4838	0.4318	0.6164	0.5007	0.3848	0.5585	0.4492
+FF (Feature Fusion)	0.4328	**0**.**6296**	0.5066	0.4467	0.5998	0.5085	0.3928	0.5813	0.4636
+POS+FF	0.4363	0.5957	0.5024	0.4353	0.6171	0.5051	0.3994	0.5786	0.4660
+NER+FF	0.4471	0.6114	0.5072	**0**.**4471**	0.6114	0.5072	**0**.**4053**	0.5795	0.4678
Full model (BioBERT+POS+NER+FF)	**0**.**4517**	0.6294	**0**.**5197**	0.4444	**0**.**6419**	**0**.**5165**	0.3937	**0**.**6098**	**0**.**4688**

Bold values represent the highest results

**Table 4:** Robustness detection experiment results using the average evaluation value and the standard deviation among different seeds (12345, 24, 488)

Model	6b Factoid QA		
	SAcc	LAcc	MRR
BioBERT (main baseline)	0.4048 ± 0.0107	**0**.**6278** ± **0**.**0061**	0.4927 ± 0.0102
Our Model (BioBERT+POS+NER+FF)	**0**.**4325** ± **0**.**0167**	0.6200 ± 0.0138	**0**.**5063** ± **0**.**0137**

Model	7b Factoid QA		
	SAcc	LAcc	MRR
BioBERT (main baseline)	**0**.**4362** ± **0**.**0087**	0.6146 ± 0.0121	0.5059 ± 0.0045
Our Model (BioBERT+POS+NER+FF)	0.4359 ± 0.0078	**0**.**6379** ± **0**.**0035**	**0**.**5122** ± **0**.**0037**

Model	8b Factoid QA		
	SAcc	LAcc	MRR
BioBERT (main baseline)	0.3859 ± 0.0087	0.5566 ± 0.0061	0.4509 ± 0.0065
Our Model (BioBERT+POS+NER+FF)	**0**.**3916** ± **0**.**0033**	**0**.**5898** ± **0**.**0156**	**0**.**4652** ± **0**.**0040**

Bold values represent the highest results

**Table 5:** Contrast experiment results between different pre-trained language models and different external features

Model	6b Factoid QA			7b Factoid QA			8b Factoid QA		
	SAcc	LAcc	MRR	SAcc	LAcc	MRR	SAcc	LAcc	MRR
SciBERT	0.3688	0.5974	0.4544	0.4203	0.6051	0.4919	0.3793	0.5737	0.4496
SciBERT+POS+NER	0.3967	0.5959	0.4766	0.4253	0.5901	0.4900	0.3874	0.5523	0.4499
Baseline (BioBERT)	0.3973	0.6217	0.4838	0.4318	0.6164	0.5007	0.3848	0.5585	0.4492
BioBERT+BioNER+FF	0.4380	0.6075	0.5078	0.4355	0.6113	0.5012	0.3982	0.5875	**0**.**4692**
BioBERT+POS+BioNER+FF	0.4171	0.6263	0.5011	0.4419	0.6211	0.5106	**0**.**4032**	0.5858	0.4689
Our model (BioBERT+POS+NER+FF)	**0**.**4517**	**0**.**6294**	**0**.**5197**	**0**.**4444**	**0**.**6419**	**0**.**5165**	0.3937	**0**.**6098**	0.4688

Bold values represent the highest results

### Experimental results and analysis

For each factoid question, it is required to return 5 best matched answer spans extracted from one or multiple given passages in order. We employed three official metrics used by BioASQ challenge, strict accuracy (SAcc), lenient accuracy (LAcc) and mean reciprocal rank (MRR), to evaluate the result, of which SAcc measures the strict answer location capability, LAcc measures the model’s perception of answers range, and MRR reflects the overall quality of the returned answers [25]:

10 $$\begin{aligned} SAcc= & \, \frac{C_{1}}{n} \end{aligned}$$

11 $$\begin{aligned} LAcc= & \, \frac{C_{5}}{n} \end{aligned}$$

12 $$\begin{aligned} MRR= & \, \frac{1}{n}\sum _{i=1}^{n}\frac{1}{r(i)} \end{aligned}$$

where *n* is the test set size; $C_{1}$ represents the number of factoid questions correctly answered by the first returned answer span, while $C_{5}$ is the number of questions that have been correctly answered considering the whole five returned answers, and r(i) is the rank of golden answer among the five returned answer spans for each question *i*. In situation that golden answer of question *j* does not occur among returned spans, we considerate *r*(*j*) as infinite and $\frac{1}{r(j)}$ as 0. We have leveraged the official tools provided in the BioASQ web site to evaluate our experimental results [25].

We conducted several different experiments and evaluated our model on BioASQ 6b, 7b and 8b test sets. The results are shown as following. Table 2 shows the comparison results of our model and different baseline models on BioASQ 6b, 7b and 8b challenges, where the comparative results were collected from the related papers and BioASQ website^4^. In particular, for the baselines’ results of 8b challenge, considering the consistency of the model’s performance, we selected the best models that participated in all five batch competitions to compare. The chosen models in this research are historical participants with excellent results in the BioASQ challenge:

- *AUTH* [29] Participating in BioASQ 6b and 7b tasks, AUTH model utilized word embedding as textual representations directly and extracted some external biomedical features based on MetaMaps, BeCAS, and WordNet to enhance model’s performance;
- *ZhuLab-Fudan* [30] ZhuLab system adopted both traditional information retrieval approaches and knowledge-graph based method to conduct factoid question answering task in BioASQ 6b and 7b challenges; in 8b challenge, they experimented with different pre-trained language models, such as BERT [5], BioBERT [7], XLNet [13] and SpanBERT [33], combining with transfer learning and voting method [34], to better solve biomedical factoid QA task.
- *Google* [31] Based on BERT pre-trained language model [5], Hosein et al. firstly fine-tuned QA model on two general QA datasets NQ [35] and CoQA [36] and then completed domain QA task;
- *BioBERT* [11] BioBERT was based on pre-trained language model BERT [5] as well but further retrained on a large-scale biomedical corpus. After continuously fine-tuning on SQuAD1.0 and BioASQ training sets, it achieved a remarkable improvement in BioASQ 6b test set and won the first place in 7b challenge;
- *UNCC* [12] Based on domain pre-trained language model BioBERT, UNCC fine-tuned the model firstly on SQuAD2.0 [37] and added biomedical lexical answer type as additional features;
- *Umass* [32] Based on BioBERT PLM, Umass team introduced a biomedical entity de-noising task in the pre-training process to help the PLM learn a better domain text representation;
- *KU-DMIS-2020* [14] Jeong et al. proposed using MultiNLI dataset [38] and natural language inference (NLI) tasks to enhance BioBERT’s performance for domain QA tasks, which gained excellent results on BioASQ 8b challenge.

Following the same fine-tuning process as the main baseline model BioBERT, our model was initialized with BioBERT PLM and leveraged POS, NER these two external features as additional information on both general QA training set (SQuAD 1.0) and biomedical training process under proposed framework shown in Fig. 1. In our experiments, we have noticed an improvement of all of three metrics(SAcc, LAcc, and MRR) by our model, and achieved a SOTA result for all metrics on 6b, 8b test sets and two metrics on 7b test set, which demonstrated that our feature-enriched structure could indeed improve biomedical question answering task’s performance.

Besides, we took several ablation experiments as well to prove the importance of both POS and NER features. All experiments are implemented under the same seed and hyper-parameters. Experimental results are shown in Table 3, where the base model is the BioBERT model. FF model is the base model simply added by an encoder layer, which can eliminate the influence of a deeper neural network structure in the feature fusion module. We also verified the effectiveness of POS and NER features, respectively. The results show that the addition of a single feature makes the experimental results unstable and sometimes even leads to a side effect, and the combination of all of these modules could achieve the best performance. We will further discuss this phenomenon with concrete examples in the “Discussion” section.

To further explore the framework’s stability and execute the error analysis of the model’s performance, we randomly selected several different seeds for repeated experiments on 6b, 7b and 8b three data sets. Shown in Table 4, the experimental results slightly fluctuated on SAcc and LAcc two metrics, while the overall indicator MRR showed a better performance and proved the effectiveness of our method. Overall, the baseline model BioBERT and our model have similar standard deviations in multiple experiments.

Furthermore, we also conducted several comparative experiments to demonstrate the proposed general features’ effectiveness and the overall framework’s reasonability, of which the results are shown in Table 5. On the one hand, we replaced BioBERT pre-trained language model utilized in our model with SciBERT, another scientific domain PLM, and experimented on BioASQ 6b, 7b and 8b, proving the effectiveness of the proposed framework. Although SciBERT was also retrained on the biomedical corpus, it is demonstrated that its overall performance is worse than BioBERT. On the other hand, we compared the selected general features with biomedical domain features. In our experiments, we leveraged BERN [27] as the BioNER annotator, which could recognize seven different categories of biomedical named-entity. Similarly, implemented under the same hyper-parameters, it is demonstrated in Fig. 5 that our proposed framework and the selected general features can better improve the performance of biomedical QA task. Remarkably, as the training dataset in the biomedical domain expands and the volume of data increases, the role of domain named entities is gradually amplified under our proposed framework, which reflects the importance of data in the neural network models and the effectiveness of our feature fusion method. The results also remind us that as the biomedical domain community grows and domain labeled data increase, we should pay more attention to domain features and taggers.

**Fig. 2:**
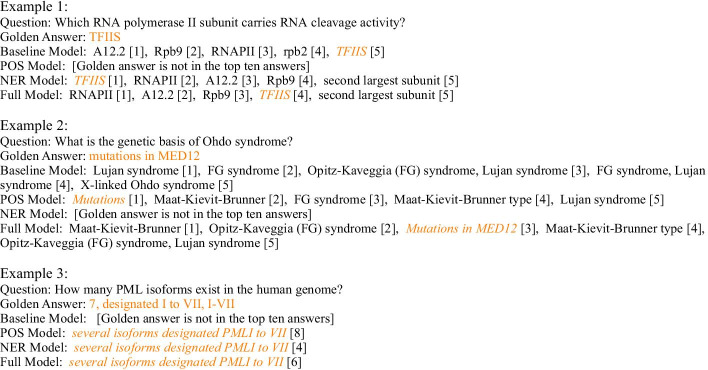
Biomedical QA case study—QA examples. Three examples are extracted from BioASQ challenge test set. Baseline model is the BioBERT Model; POS Model is baseline model plus POS feature and FF (Feature Fusion) module; NER Model is baseline model plus NER feature and FF module; Full Model is baseline model plus NER, POS features and FF module. The orange text represents the correct answer, and the number in brackets represents the probability ranking of the extracted answer

**Fig. 3:**
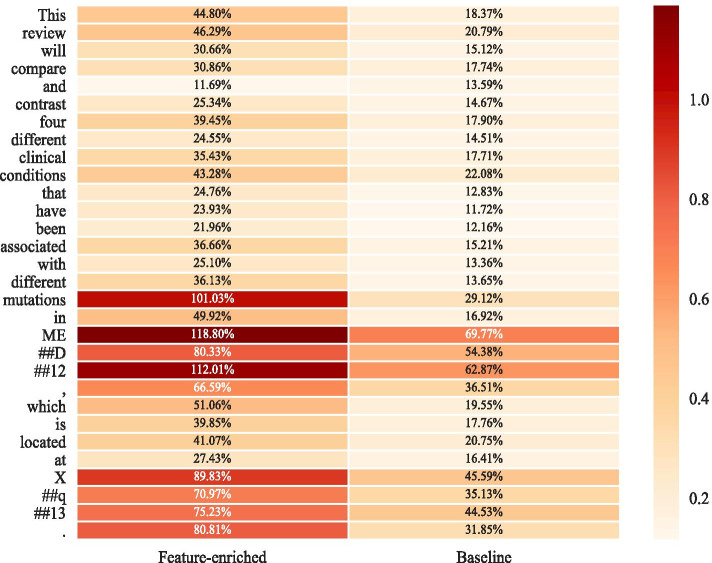
Biomedical QA case study—answer extraction Heatmap. The left side is the result of our model (BioBERT plus NER, POS features based on Feature Fusion Module), and the right side is the result of the baseline (BioBERT) model. The darker the color, the more likely this location is predicted to be the answer boundary

## Discussion

**Fig. 4:**
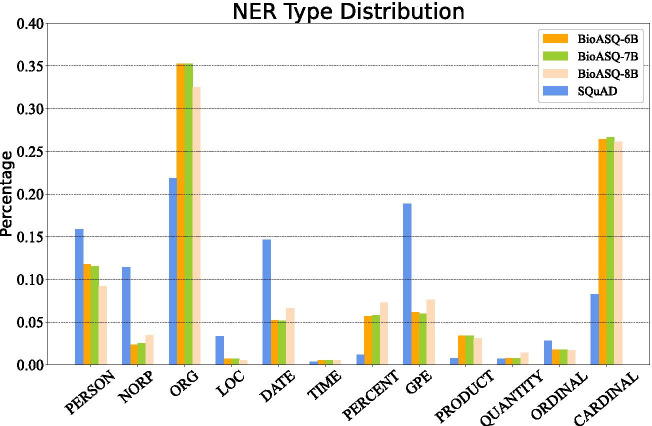
NER statistical information among four datasets. The figure shows the distribution information of the extracted NER (Named Entity) features, where for example, “ORG” stands for entities such as companies, agencies and institutions; “CARDINAL” represents general numeral entities; “GPE” stands for entities including countries, cities and states

**Fig. 5:**
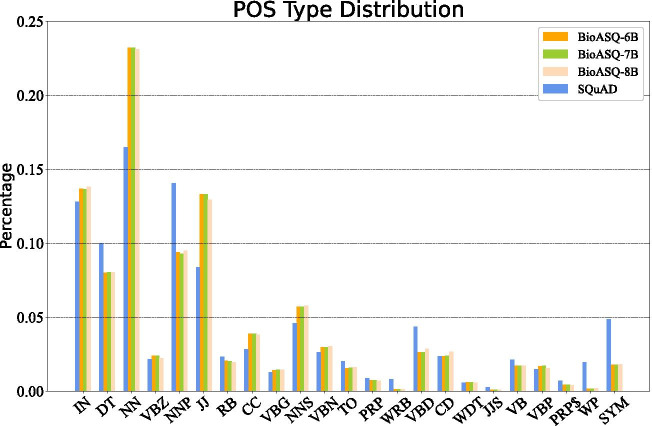
POS statistical information among four datasets. The figure shows the distribution information of the extracted POS (Part-Of-Speech) features, where for example, “NN” is the tag of the noun, “JJ” stands for adjective, and “IN” represents preposition and subordinating conjunction

**Fig. 6:**
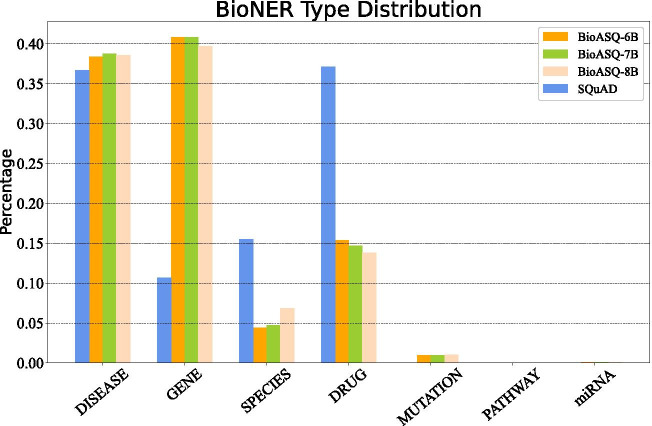
Biomedical NER statistical information among four datasets. The figure shows the distribution information of the extracted Biomedical NER (Named Entity) features

**Table 6:** Statistical analysis of unanswerable factoid questions

Title		Batch 1 (%)	Batch 2 (%)	Batch 3 (%)	Batch 4 (%)	Batch 5 (%)	All (%)
6b	Weakly unanswerable ratio	9.7	4.8	9.4	18.2	18.2	13.0
	Strongly unanswerable ratio	12.9	9.5	12.5	9.1	15.9	12.4
	All unanswerable ratio	22.6	14.3	21.9	27.3	34.1	**25.4**
7b	Weakly unanswerable ratio	17.9	16.0	10.3	5.9	17.1	13.6
	Strongly unanswerable ratio	12.8	4.0	13.8	5.9	5.7	8.6
	All unanswerable ratio	30.7	20.0	24.1	11.8	22.8	**22.2**
8b	Weakly unanswerable ratio	3.1	8.0	17.9	2.9	9.4	7.9
	Strongly unanswerable ratio	18.8	16.0	14.3	14.7	6.2	13.8
	All unanswerable ratio	21.9	24.0	32.2	17.6	15.6	**21.7**

Bold values represent the overall percentage of unanswerable questions

### Case study

Here we introduce some test cases to analyze the concrete influence of two external features added in our models in an intuitive way, as shown in Fig. 2, where the text in orange represents the correct answer, and the number in brackets represents the probability ranking of the extracted answer. For the first instance, the expected answer is a biomedical proper noun, and it is recognized as an ORG entity by NER tool. Although the entity tag is meaningless for a transcription factor TFIIS, it locates and emphasizes the boundary of this entity, which can supply extra information for our model. We can notice that the NER model provides the best performance in the first example. In this example, POS features would not have been practical, and the introduction of such syntactic structure even reduced the sensitivity of the model to the true answer to some extent, which also confirms the instability of a single feature shown in Table 3.

Besides, sometimes paying too much attention to an entity can misdirect our network and lead to a wrong answer type. In the second case, the expected answer is a genetic action, but both the baseline model and the NER model focus too much on “syndrome”; instead, the POS model which introduces syntactic features can correctly identify the expected answer type. Besides, from the Fig. 3 where the depth of the color represents the possibility that the token is selected as one of the answer boundaries, we can notice more intuitively that our feature-enriched model can distinct better both biomedical named-entity (Xq13) and the expected answer (mutations in MED12).

The third instance shows a trade-off among POS, NER and the full model when the question involves biomedical entity, where the baseline model can not detect the target answer span even considering the first ten returned answers while our feature enriched models can catch the required number information indeed and ameliorate model’s performance in different degree.

### POS and NER analysis

We analyzed statistical information and performance of the extracted features in detail. The statistic distribution of POS and NER tags among four training datasets are shown as Figs. 4 and 5. We can notice that the POS distribution in biomedical corpus is similar to that on SQuAD, however, the NER distributions have a great difference. As we leveraged a general name entity recognition tool and the expected entity classes are mainly companies/agencies/institutions (ORG), countries/cities/states (GPE), non-GPE locations (LOC), date, times, and other numeral words or phrases, these general named entity tags marked on biomedical corpora do not have strong semantic significance, but they remain the consistency with the general corpus and simultaneously play an important role in entity boundary distinction and entity classification.

In addition, in order to compare with the chosen general NER features, we also implemented our experiments with biomedical NER, of which the distribution information among four datasets is shown in Fig. 6. With fewer types of entities, we could notice a great distribution difference in GENE, SPECIES and DRUG these three classes between general and biomedical domain data. According to the number of marked NER tags, biomedical NER could indeed enrich biomedical domain lexical information; however, it could only provide limited external information on the SQuAD dataset and its performance improvement for QA tasks under our framework is weak and unstable when the domain dataset size is small. As mentioned earlier in the “Results” section, it is noteworthy that the role of biomedical domain entity features becomes more and more manifest as the domain training data increases. In the future, with the emergence of more labelled biomedical QA data, we should probably consider more on how to better utilize the domain annotators and incorporate the domain features such as BioNER into the model to improve the performance of QA tasks further. Besides, as the amount of biomedical domain QA training data becomes more balanced with general domain training data, we could also consider using domain-specific POS taggers such as MedPost [39] and scispaCy [40], which were trained on the biomedical domain corpus, to better capture the structural features of domain text and to improve task performance.

### Unanswerable questions

Our model for factoid question answering is mainly based on extractive machine reading comprehension, which means the golden answer can always be continuously extracted from the given passage. However, after analyzing the BioASQ test data concretely, we found some “Unanswerable Questions” that can not be directly answered by the given contexts, ignoring the case difference. Further divided into two sub-categories, weakly unanswerable and strongly unanswerable, these questions’ statistical information is shown in Table 6. Among them, “weakly unanswerable” means that similar answers can be extracted from the given text and can be equated with the golden answer after lexical transformation, singular-plural transformation, abbreviation reduction, special symbol processing, phrase structure changes, etc. For example, for question “Which phosphatase is inhibited by LB-100?” in batch 1 of 8b test data, the given context is “Here, we examined radiosensitizing effects of LB-100, a novel inhibitor of PP2A against AAM as a novel treatment strategy”, and the golden answer is “Protein phosphatase 2A”. From the given context, we could only extract “PP2A”, the abbreviation of the correct answer. In such cases, the text fragment returned by direct extraction is equivalent to the golden answer at the semantic and knowledge level, only the representation of the text is different, and some regularized or artificial changes can obtain the target answer.

As for the “strongly unanswerable” questions, golden answers usually cannot be obtained by single-point extraction, and often need to be extracted at multiple places in the given passages, spliced and summarized from the extracted fragments. There are some answers that even require the common sense and domain knowledge and could be obtained only by generative models. Answering these questions involves more inference, numeric calculation, multi-passage question answering, and text generation technologies, which is beyond our current model’s capabilities.

## Conclusions

In this work, we leveraged external textual features to improve the QA text’s matching degree for biomedical question answering task. We adopted general syntactic and lexical features such as POS and NER to improve the QA matching degree, emphasize the biomedical sentence structure and proper entity, and bridge the gap between general and biomedical corpus. Besides, we proposed a novel framework to merge these features into the pre-trained language model in order to enhance downstream QA task performance. The results of experimental studies on BioASQ challenges have shown that the proposed method can achieve satisfying performance.

In the future, on the one hand, for those unanswerable questions in BioASQ challenge, we will further study how to introduce inference and generation modules in our framework to better answer these questions and complete machine reading comprehension task in the biomedical domain; on the other hand, we would like to further analyze the role of external features in terms of model interpretability at the theoretical and experimental levels, to utilize both the general and the biomedical domain features better. Besides, the work will also be developed to better merge external features, as an enhancement of knowledge detection and representation, into a pre-trained language model to improve the model’s performance on cross-domain natural language processing understanding and generation tasks.

## Data Availability

The source code and trained models are available at https://github.com/xugezheng/BioQAExternalFeatures. The SQuADv1.1 dataset utilized in this study is available at https://datarepository.wolframcloud.com/resources/SQuAD-v1.1. The data of BioASQ factoid Question Answering task is available from the BioASQ challenges in the official website, http://participants-area.bioasq.org/datasets/ [25].

## Footnotes

1 https://github.com/dmis-lab/bioasq-biobert

2 https://github.com/dmis-lab/bioasq8b

3 https://datarepository.wolframcloud.com/resources/SQuAD-v1.1

4 http://participants-area.bioasq.org/results/8b/phaseB/
